# Schisandrin B for the treatment of male infertility

**DOI:** 10.1002/ctm2.333

**Published:** 2021-02-23

**Authors:** Di‐Xin Zou, Xue‐Dan Meng, Ying Xie, Rui Liu, Jia‐Lun Duan, Chun‐Jie Bao, Yi‐Xuan Liu, Ya‐Fei Du, Jia‐Rui Xu, Qian Luo, Chong‐jun Zhao, Zhan Zhang, Shuang Ma, Wei‐Peng Yang, Rui‐Chao Lin, Wan‐Liang Lu

**Affiliations:** ^1^ State Key Laboratory of Natural and Biomimetic Drugs, Beijing Key Laboratory of Maolecular Pharmaceutics and New Drug System, and Scool of Pharmaceutical Sciences Peking University Beijing 100191 China; ^2^ Beijing Key Laboratory for Quality Evaluation of Chinese Materia Medica, School of Chinese Materia Medica Beijing University of Chinese Medicine Beijing 100102 China; ^3^ Institute of Chinese Materia Medica China Academy of Chinese Medical Science Beijing 100700 China

Dear Editor,

The decline of male fertility and its consequences on human populations are severe public‐health issues, and oligoasthenospermia is a common cause of male infertility.^1^, ^2^However, the treatment choices for male infertility are limited.^3^ Here, we first report that schisandrin B (SB) was screened from Wuzi Yanzong‐Pill (WP), which enabled the treatment of male infertility, and uncover the underlying mechanism.

The ancient prescription WP has been widely used for treating oligoasthenospermia since the Tang dynasty of China. However, its active component(s) are still not clear. To find active component(s), we had identified 106 major compounds in WP using UPLC‐ESI‐LTQ‐Orbitrap‐MS,^4^ and their similarity scores of drug molecular structures were evaluated using MedChem Studio, 22 compounds have higher similarity scores of drug molecular structures, and subsequently, the relative abundances of 22 components were assessed by factor analysis with SPSS (Figure 1A and B; Supplementary Dataset S1–S3). SB had the highest comprehensive score. To determine its oral availability according to site of action, 3 h after oral administration SB, SB was identified in the plasma and testicular tissues of normal male mice (Figure 1C–I; Figure S1), demonstrating the SB availability in plasma and testicular tissue of mice upon oral administration. Different drugs used to treat a disease usually produce similar gene‐profiling signatures,^5^ and to verify whether SB had testicular gene (TG) expression similar to WP, we investigated SB involvement in the regulation of TG expression by comparing it with that of WP in an established model of oligoasthenospermia mice (OM).^6^ In mice, the expression of 100 of the most upregulated and downregulated TGs (50:50) by WP was compared with the corresponding TGs regulated by SB. Both heatmap and Pearson's correlation analysis revealed that SB and WP had similar TGs signature and were highly correlated (*r*  = 0.735) ( Figure 1J and K; Supplementary Dataset S4), suggesting that SB could be used to treat male infertility.

**FIGURE 1:**
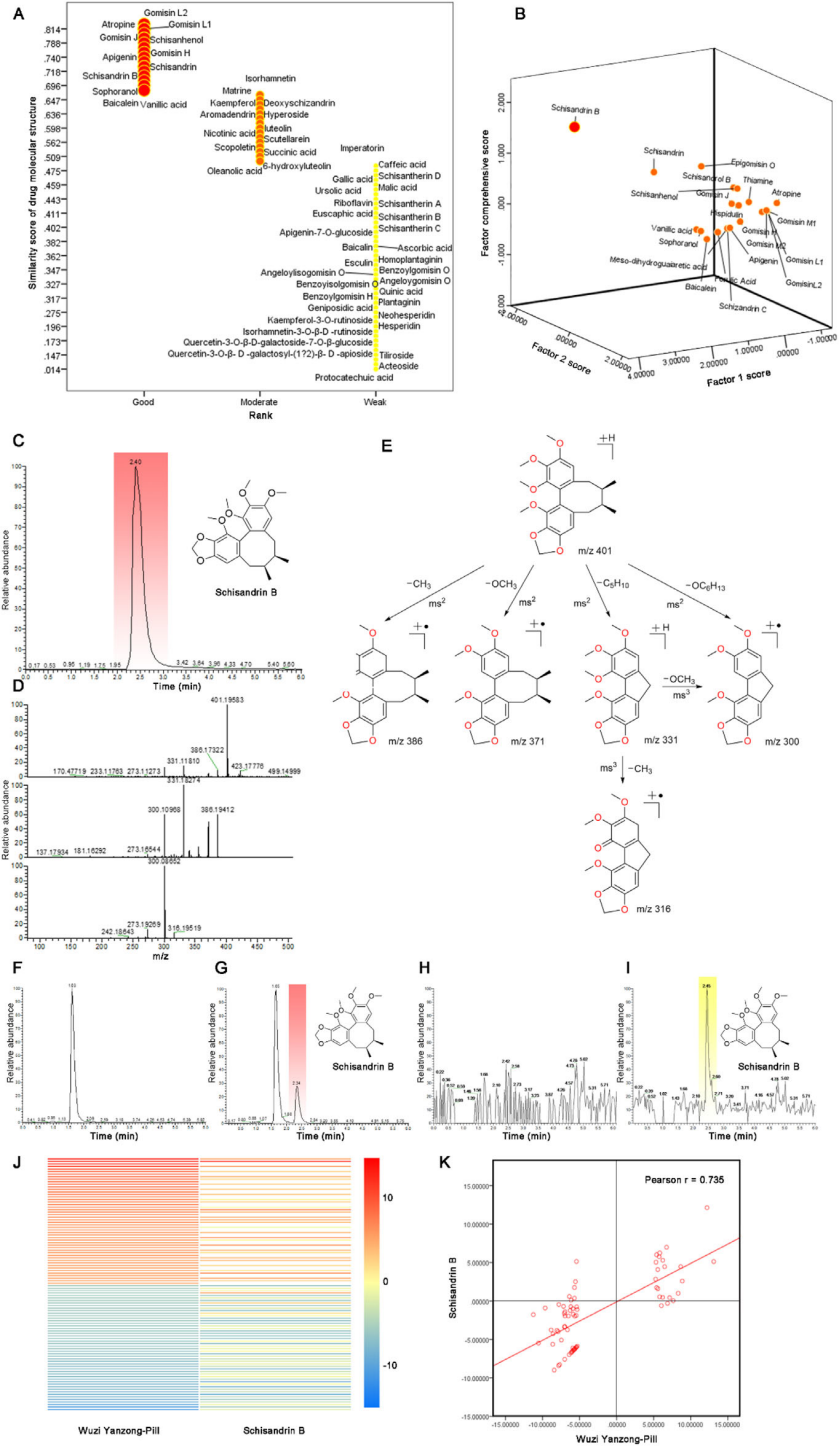
Schisandrin B is identified as a potent agent for the treatment of male fertility. *Notes*: The studies (A and B) were performed by simulation and statistical analyses in accordance with measurements on the methanol extract of Wuzi Yanzong‐Pill (WP) by UPLC‐ESI‐LTQ‐Orbitrap‐MS. (A) Similarity scores of drug molecular structures for 106 major compounds extracted from WP. The study was performed for evaluating the druggability for each component by software of Medchem Studio v3.0 (Simulations Plus, Inc., Lancaster, CA). The result reveals that schisandrin B (SB) along with 21 other components has been listed in the higher score in evaluating the similarity scores of drug molecular structures. (B) Factor comprehensive score of SB among 22 compounds which have higher similarity scores of drug molecular structures. The study was performed for further screening the drug candidate with the Factor Analysis with software of SPSS v 20 (IBM, Armonk, NY). Factor 1, the similarity scores of drug molecular structures; Factor 2, the relative abundances of a compound among 22 compounds extracted from WP. The result indicates that SB has the highest druggability among them in evaluating the factor comprehensive score. The studies (C–I) were analyzed by UPLC‐ESI‐LTQ‐Orbitrap‐MS: (A) typical total ion chromatogram (TIC) of pure SB; (B) triple fragment spectra of pure SB; (C) fragmentation pathways of SB; (D) typical TIC chromatogram of blank mouse plasma; (E) typical TIC chromatogram of mouse plasma after oral administration of SB (20 mg/kg) at 3 h; (F) typical TIC chromatogram of blank mouse testis; (G) typical TIC chromatograms of mouse testis after oral administration of SB (20 mg/kg) at 3 h. The studies (J–K) were performed by gene sequence profiling on the testicular samples of oligoasthenospermia mice (OM) after oral administration of SB (20 mg/kg/day for 2 weeks; *n* = 3) or WP (1.56 g/kg/day for 2 weeks; *n* = 3): (H) gene heatmaps for the most significant up and downregulated genes (each 50 genes) in the testicular samples from OM after oral treatment with WP (1.56 g/kg/day for 2 weeks; *n* = 3) or SB (20 mg/kg/day for 2 weeks; *n* = 3). Red color indicates the upregulated genes; blue color indicates the downregulated genes. (I) Pearson correlation of the regulated gene log‐folds between WP and SB. *r* represents correlation coefficient

To observe the spermatogenic effect of SB, testicular tissues and sperm samples from OM were sampled after oral administration at 2 week, SB had similar spermatogenic effects to WP, and could repair damaged seminiferous tubules and spermatogenic cells in OM testis (Figure 2A). Moreover, SB and WP could increase the sperm number (concentration), sperm‐activity (sperm mobility and progressive mobile sperm), sperm‐motion velocities (curvilinear velocity, straight‐line velocity, and average path velocity), and improve sperm‐motion parameter (straightness, beat cross frequency, amplitude of lateral head displacement, and linearity) (Figure 2B–D; Videos S1–S, 5; Supplementary Dataset S5). Furthermore, to investigate the reproductive ability, normal mice, OM, OM after treatment with SB or WP for 2 weeks and male mice were mated with female mice at a 1:2 ratio, respectively. Treatment of OM with SB or WP increased the number of pups in the first litter and average number of births, showing fertility close to that of normal mice, respectively (Figure 2E; Supplementary Dataset S6).

**FIGURE 2:**
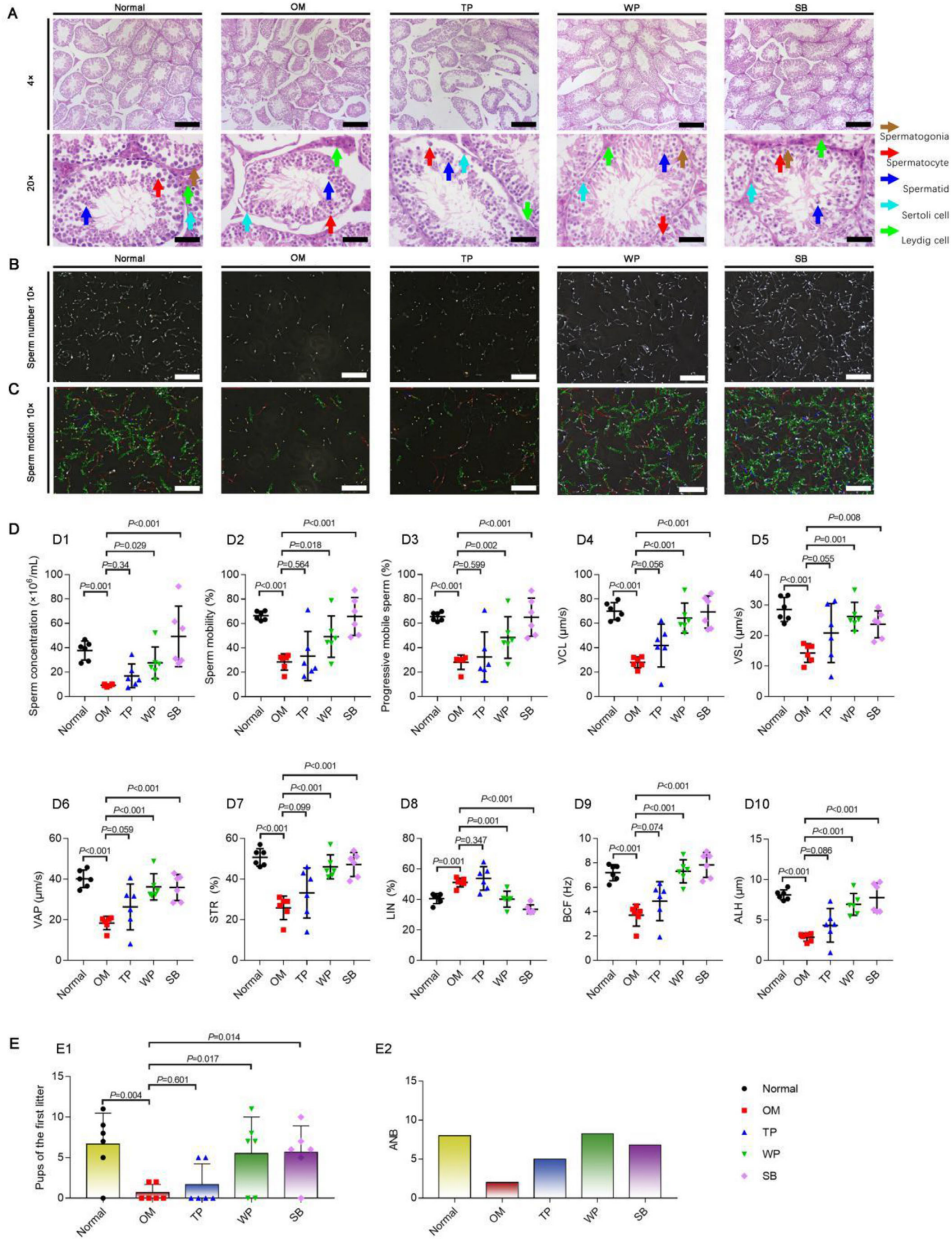
Schisandrin B enables to enhance male fertility in oligoasthenospermia mice. *Notes*: The studies (A–C) were performed to verify the efficacy of SB in treatment of male fertility, including spermatogenesis, sperm number, and sperm activity in Balb/c mice. (A) Hematoxylin and eosin staining images of mouse testicular samples. The samples were obtained from normal mice (*n* = 6), OM (*n* = 6), and TP‐treated OM (*n* = 6; *i.p*.TP 0.2 mg/kg /twice a week for 2 weeks), WP‐treated OM (*n* = 6; *i.g*. WP 1.56 g/kg/day for 2 weeks), or SB‐treated OM (*n* = 6; *i.g*. SB 20 mg/kg/day for 2 weeks). *i.g*., intragastric administration; OM, oligoasthenospermia mice; SB, schisandrin B; TP, testosterone propionate; WP, Wuzi Yanzong‐Pill. Scale bar, 200 μm. Brown arrow indicates spermatogonia; red arrow indicates spermatocyte; blue arrow indicates spermatid; cyan arrow indicates Sertoli cells; and green arrow indicates leydig cells. The results demonstrate that SB enables to repair the disrupted spermatogenesis of OM. (B) Sperm number images of mouse cauda epididymidis samples under Suiplus Semen Analysis Automatic Detection System (Suiplus, BeiJing, China). The samples were obtained from normal mice (*n* = 6), OM (*n* = 6), and TP‐treated OM (*n* = 6; *i.p*. TP 0.2 mg/kg/twice a week for 2 weeks), WP‐treated OM (*n* = 6; *i.g*. WP 1.56 g/kg/day for 2 weeks), or SB‐treated OM (*n* = 6; *i.g*. SB 20 mg/kg/day for 2 weeks). The results directly demonstrate that SB enables to increase the sperm number of OM. The dynamic videos of this study are available in Videos S1–S5. (C) Sperm motion track images of mouse cauda epididymidis samples under Suiplus Semen Analysis Automatic Detection System (Suiplus). The samples were obtained from the same as above (Figure 2B). The observation displays that SB increases the sperm mobile activity of OM. The analyses were performed for evaluating the quality of sperms in OM after oral treatment with SB. (D) Quality of spermatogenesis. D1, sperm concentrations; D2, sperm mobility; D3, progressive mobile sperms; D4, curvilinear velocity (VCL); D5, straight‐line velocity (VSL); D6, average path velocity (VAP); D7, straightness (STR); D8, linearity (LIN); D9, beat cross frequency (BCF); D10, amplitude of lateral head displacement (ALH). The studies (E and F) were performed for evaluating the male reproductive ability by comparing the number of pups in the first litter of female mice, and the average number of births (ANB; = total number of births/birth females). Each male mouse was placed in one cage, and mated with two females. (E) Efficacy in enhancing reproductive ability (*n* = 3). E1, pups in the first litter of female mice; E2, average number of births (ANB; = total number of births/birth females). These data demonstrate that SB significantly increases male reproductive ability, leading to an enhanced ability of male mice to make female mice pregnant and the mean number of offspring

We wished to reveal the mechanism of action of SB. Hence, RNA sequencing was done on the testicular tissues of OM after SB treatment for 2 weeks. SB could alter substantial TGs (2033) (Figure 3A), and directly regulated three reproductive pathways: gamete generation, meiotic cell cycle, and spermatid development, and enriched 137 TGs in the three pathways (Figure 3B and C; Supplementary Dataset S7 and S8). Among 137 TGs, *Fst* showed the remarkable upregulation of expression upon oral administration of SB (Supplementary Dataset S8). Based on follistatin, protein (encoded by *Fst*) promotes the growth and development of spermatogenic cells by blocking the action of activin‐A protein.^7^ Overexpression of activin‐A protein (encoded by *Inhba*) can induce apoptosis of spermatogenic cells and lead to spermatogenic blockage, and downregulation of *Inhba* expression could contribute directly to the decrease in activin A‐expression, thereby attenuating spermatogenic blockage.^8^ Upregulated *Fst* expression would increase follistatin expression, which enables the blockade of the action of overexpressed activin‐A, thereby repairing spermatogenic blockage.^9^ Therefore, we further investigated the expression of *Inhba* in testicular tissues, and *Inhba* expression was downregulated markedly in the testicular tissue of OM after SB treatment (Figure 3D, Supplementary Dataset S9). Furthermore, the upregulation of *Fst* expression and downregulation of *Inhba* expression were verified by RT‐qPCR (Figure 3E, Supplementary Dataset S10). These results indicated that SB could treat OM by regulating the expression of *Fst* and *Inhba*.

**FIGURE 3:**
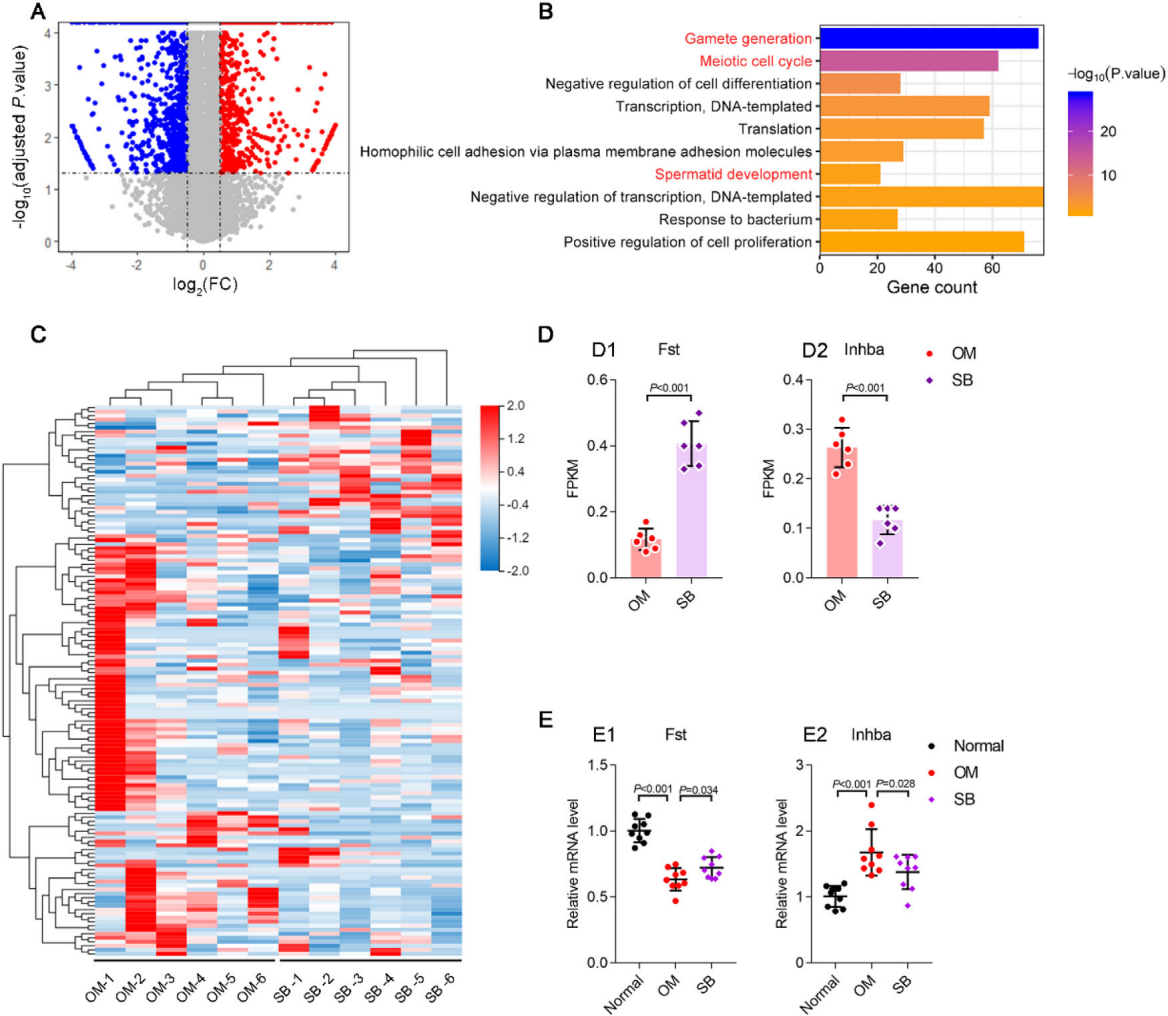
Schisandrin B regulates testicular gene expressions of *Fst* and *Inhba* in the reproductive pathway. *Notes*: The studies (A–D) were performed to reveal the regulated functional genes by schisandrin B (SB) by using gene sequencing on testicular samples of oligoasthenospermia mice (OM) and on those of SB‐treated OM (*i.g*. SB 20 mg/kg/day for 2 weeks; *n* = 6): (A) Volcano plot of SB‐mediated changes of testicular genes by software of Dr Tom (v2.0, Beijing Genomics Institute, BGI Shenzhen, China). The samples were obtained from OM (*n* = 6) and SB‐treated OM (*i.g*. SB 20 mg/kg/day for 2 weeks; *n* = 6). SB‐changed genes were identified with two threshold criteria: fold up or downregulation in SB‐treated mice of |log_2_^(FC)^| > 0.58, and adjusted *p* value of less than 0.05. The results reveal that after oral administration of SB in OM, it significantly upregulates 836 genes, while it downregulates 1197 genes. (B) Top 10 GO pathways involved in above changed genes by GO analysis. GO enrichment was performed on above regulated‐genes (totally 2033 genes) by using software of Dr Tom. The results indicate that, among top 10 GO pathways, three reproductive pathways, including gamete generation, meiotic cell cycle, and spermatid development, are involved in the gene regulations by SB. Besides, a number of 137 genes are included in the reproductive pathways. (C) Gene heatmap for SB‐regulated testicular genes (*n* = 137 genes) by using software of Dr Tom. The results indicate that oral administration of SB significantly alters testicular gene signature in OM. Furthermore, it reveals that *Fst* gene is the mostly regulated functional gene in viewing the absolute fold change or adjusted *p* value. (D) *Fst* and *Inhba* gene expressions in testicular samples of OM after oral treatment of SB. D1, *Fst* gene expression level; D2, *Inhba* gene expression level. FPKM represents the fragments per kilobase per million mapped fragments. The results reveal that SB significantly upregulates *Fst* gene, while downregulates *Inhba* gene in OM after oral treatment of SB. The studies were performed for verifying the regulated mRNA levels of *Fst* and *Inhba* gene expressions by RT‐qPCR in testicular samples of OM after oral treatment of SB. (E) mRNA levels of *Fst* and *Inhba* in testicular samples of OM after oral treatment of SB. E1, *Fst* mRNA expression; E2, *Inhba* mRNA expression. The samples were obtained from normal mice (*n* = 3), OM (*n* = 3), and SB treated‐OM (*i.g*. SB 20 mg/kg/day for 2 weeks; *n* = 3). The results exhibit that oral treatment of SB significantly increases *Fst* mRNA expression, while decreases *Inhba* mRNA expression in testicular tissue of OM, indicating that SB could treat oligoasthenospermia by regulating expressions of *Fst* and *Inhba* genes

With regard to potential clinical use, we investigated the plasma and testicular pharmacokinetics of SB in normal mice after oral administration using UPLC‐QqQ‐MS/MS. The measurement was validated^10^ and consisted of specificity, calibration curves, correlation coefficients, linear ranges, and lower limit of quantifications; intra/interday precisions and accuracies; recovery stability; and measurement stability (Figure S2A–C, E; Supplementary Dataset S11–S14). After oral administration, plasma and testicular concentration‐time profiles for SB were plotted (Figure S2D and F; Supplementary Dataset S15 and S16), and the corresponding pharmacokinetic parameters were calculated (Figure S2G and H; Supplementary Dataset S17 and S18). Plasma parameters demonstrated that oral administration led to rapid absorption and an effective exposure of SB in blood, and SB could be eliminated from blood within 1 day (seven‐fold half‐life washing‐out period about 21 h). After absorption, SB was distributed effectively into testicular tissue but with a delay, and SB in testicular tissue had comparable pharmacokinetic behavior to that in blood. These results revealed that SB could be absorbed rapidly after oral administration, and became fully available at the intended action site, indicating a remarkable potential for clinical application.

In conclusion, SB as an active component was screened from WP, which enabled the repairs of spermatogenesis arrest and male infertility. The action mechanism could be explained by the repaired spermatogenesis via upregulation of *Fst*, while downregulation of *Inhba* genes involved in the reproductive signaling pathway. The encouraging preclinical data with pharmacokinetics warranted a rapid development of this new class of therapeutic agent. Our study provides a promising drug for treatment of male infertility and a novel strategy for discovery of new small‐molecule drugs from vast plant‐based medicinal resources.

## FUNDING

This work was supported by the Beijing Natural Science Foundation (7181004), National Chinese Medicine Standardized Project of China (ZYBZH‐C‐BJ‐03), National Natural Science Foundation of China (81760837), and in part by the National Natural Science Foundation of China (81673367 and 81874303).

## COMPETING INTERESTS

The authors declare no competing interests in relation to publication of this study.

## AUTHOR CONTRIBUTIONS

Lu W.‐L., and Lin R.‐C. designed the study and supervised the analyses. Zou D.‐X., and Meng X.‐D. completed the major research work. Xie Y., Liu R., Duan J.‐L., Bao C.‐J., and Liu Y.‐X. undertook experiments under the direction of Lu W.‐L. and Lin R.‐C.. Du Y.‐F., Xu J.‐R., Luo Q., Zhao C.‐J., Zhang Z. , Ma S. and Yang W.‐P. helped with data analyses. Zou D.‐X., Meng X.‐D.,Lin R.‐C. and Lu W.‐L. wrote the manuscript with input from all authors. All authors approved the final version for submission.

All authors approved the final version for submission.

## Supporting information

SuppMat

FigureS1–S2

VideoS1

VideoS2

VideoS3

VideoS4

VideoS5

DatasetsS1–S18
