# Supplementary material for: Schisandrin B for the treatment of male infertility
Source: Clin Transl Med. 2021 Feb 23;11(2):e333. doi: 10.1002/ctm2.333 (PMC7901724; doi:10.1002/ctm2.333)
Supplement: Supplementary file 1 — SuppMat [file CTM2-11-e333-s001.doc]

**Supplementary Materials and Methods**

**Materials and Methods**

**Ethical approval of the study protocol**

All procedures involving the care and handing of animals were carried out with approval of the Authorities for Laboratory Animal Care of Peking University (Beijing, China; LA2018330).

**Reagents**

Busulfan was purchased from Macklin Biochemicals (Shanghai, China). Pure SB was obtained from the National Institutes for Food and Drug Control (Beijing, China; purity >98%; HPLC grade). TP (injection) was purchased from Shanghai General Pharmaceuticals (Shanghai, China). *Fructus lych, semen cuscutae* (fried), *fructus rubi*, *fructus Schisandrae chinensis* (steamed), and *semen plantaginis* (fried with salt) were from Beijing Tong Ren Tang Group (Beijing, China). Acetonitrile, methanol, ethanol, and formic acid were purchased from Fisher Scientific (Fair Lawn, NJ, USA; LC-MS grade). Water was purified by a Milli-Q™ ultraviolet purification system (Millipore, Bedford, MA, USA). Dimethyl sulfoxide was obtained from Sigma–Aldrich (Saint Louis, MO, USA). Polyethylene glycol (PEG)_400_ was purchased from Harveybio Gene Technology (Beijing, China). Sperm Culture Medium 199 (M199) was obtained from Thermo Scientific (Waltham, MA, USA). Bovine serum albumin was purchased from Solarbio (Beijing, China). PCR primers were obtained from Tsingke Biological Technology (Beijing, China). All other chemicals were from commercial sources.

**Animals**

Male and female Balb/c mice (10 weeks; 20.0 ± 2 g) were obtained from the Department of Laboratory Animal Science, Peking University Health Science Center (order ID: SCXK (jing) 2016-0010). Each animal was housed in an individual cage at controlled temperature (25±1°C) and humidity (55±5%) and exposed to a 12-h light–dark cycle (7 pm to 7am). Animals had free access to food (regular chow comprising 5% fat, 53% carbohydrate and 23% protein) and water unless indicated otherwise.

**Ancient formulation for treatment of male infertility**

The ancient formulation consisted of *Fructus lych*, *semen cuscutae* (fried), *fructus rubi*, *fructus Schisandrae chinensis* (steamed), and *semen slantaginis* (fried with salt). The medicinal materials were weighed, mixed (8:8:4:2:1, *w/w*) and crushed to powder (mesh size = 40). Then, they were immersed in a 10-fold volume of water for 1 h at 100°C. After boiling, heating was continued until the volume was reduced to fivefold volume as compared with the original one. The mixture was filtered immediately through gauze, concentrated to 1 g of crude drug per mL, and freeze-dried to become powders. Finally, refined honey (35 g) was mixed with freeze-dried powders (100 g) to make pellets of WP for experimental use.

**Extraction of compounds and prediction of druggability**

WP (250 mg) were extracted with 50 mL of methanol with the aid of ultrasound for 60 min. Extracts were centrifuged at 10,000 revolution per minute (rpm) for 15 min at 4°C. The supernatant was collected and passed through a filter (0.22 μm). The filtrate was collected for UPLC coupled with electrospray ionization-linear ion trap-Orbitrap tandem mass spectrometry (UPLC-ESI-LTQ-Orbitrap-MS) measurement. Based on chromatographic data, 106 major compounds were identified in the WP extract, including organic acids, flavonoids, phenylpropanoids, alkaloids and terpenoids. To predict the most promising drug candidate, the druggability was evaluated on the 106 compounds extracted from WP using MedChem Studio v3.0 (Simulations Plus). Compounds with a good drug-similarity score (i.e., druggability) were selected for further consideration by combination with drug contents in the extract. Based on the druggability and drug content (which was indicated in the corresponding peak relative abundance in the chromatogram of UPLC-ESI-LTQ-Orbitrap-MS), drug candidates were selected preliminarily in accordance with the highest factor comprehensive score using factor analysis employing SPSS v20.0 (IBM).

**Availability of SB by action site**

***Measurement:*** To ascertain if the drug candidate (SB) could be absorbed in blood or reach the action site, SB in plasma and testicular tissue (action site of drugs for male infertility) was measured by UPLC-MS/MS after oral administration of SB in male mice. Analyses were undertaken on a UPLC system (Acquity™ UPLC Ӏ-Class system; Waters, Milford, MA, USA) consisting of an auto-sampler, quaternary pump, and column oven. A C18 reverse-phase column (Acquity UPLC BEH, 100 × 2.1 mm, 1.7 μm, 130 Å) was used to separate samples. The mobile phase was 0.1% formic acid in water (A) and acetonitrile (B). The gradient elution was: 0 min 78% B, 1 min 78% B, 4 min 60% B, and 6 min 78% B. Samples were kept in the autosampler at 4°C until measurement. The column was maintained at 40°C, the flow rate was 0.3 mL/min, and injection volume was 5 μL. The UPLC was connected to a mass spectrometer (LTQ/Orbitrap; Thermo Scientific) *via* an ESI interface. The effluent was split at a ratio of approximately 3:1 (*v/v*) before entering the ESI source. Positive-ion mode was used, and operation parameters were: capillary voltage, 25 V; electrospray voltage, 4.0 kV; capillary temperature, 350°C; sheath gas, 30 (arbitrary units); auxiliary gas, 5 (arbitrary units); tube lens, 110 V. High-resolution full scan was used to scan samples with a resolution of 30,000 and a scanning mass range of 100 to 500 amu. Data-dependent scan was used to scan secondary and tertiary mass spectra, and the three peaks with the highest abundance in the upper MS level were selected for collision-induced fragmentation scanning. The normalized collision energy was set to 35%. To avoid many repeated data acquisitions on the same sample, dynamic exclusion was used for data collection with an exclusion duration of 60 s and the repeat count was set at 5 with a dynamic repeat time at 30 s. An external calibration for mass accuracy was carried out before the analysis. The measured masses were within 5 ppm of the theoretical masses. Data analyses were processed using a Xcaliber 2.1 workstation (Thermo Fisher Scientific). Meanwhile, pure SB (5 mg) was dissolved in 10 mL of methanol, passed through a filter (0.22 μm), and used as the reference for analyses.

***Dosing:*** Pure SB (1 mg/mL) was dissolved in a mixture of ethanol, PEG_400_ and 0.5% sodium carboxymethyl cellulose (CMC-Na) (1:1:1, *v/v/v*) for oral administration. Male mice were divided randomly into two groups of three. In the treatment group, each mouse was administered SB (20 mg/kg, i.g.). In the blank control group, each mouse was given physiologic saline (PS).

***Sampling:*** Three hours after dosing, venous blood (0.75 mL) was sampled and centrifuged at 5000 rpm for 10 min at 4°C. Then,+ 200 μL plasma was transferred, added to 600 μL of acetonitrile, vortex-mixed (120 s), and centrifuged (13,000 rpm, 10 min, 4°C) to remove proteins. The supernatant was evaporated at 25°C by a CentriVap™ centrifugal thickener (Labconco, Kansas City, MO, USA). The residues were dissolved in 200 μL of methanol, and centrifuged (13,000 rpm, 10 min, 4°C). The resultant supernatant was injected into the UPLC-MS/MS system.

The animals were sacrificed. The testicular tissues were collected on an ice plate at the same time as blood sampling. Next, they were washed with PS, drained with filter paper and weighed. PS (1:4, *w/v*) was added and the testicular tissue homogenized. One milliliter of testicular-tissue homogenate was centrifuged at 5,000 rpm for 10 min at 4°C. The supernatant (200 μL) was collected, and 400 μL of acetonitrile added, followed by vortex-mixing (120 s) and centrifugation at 13,000 rpm for 15 min at 4°C. Finally, the resultant supernatant was injected into the UPLC-MS/MS system.

**Involvement of SB in regulation of TG expression**

To investigate SB involvement in regulating TG expression by comparing it with that of WPs, OM models were induced by intraperitoneal injection of busulfan (20 mg/kg dissolved in sterile dimethyl sulfoxide). OM were divided into three groups of three, and treated once daily with PS, SB (20 mg/kg/d, i.g.) or WP (1.56 g/kg/d, dissolved in 0.5% CMC-Na). After 2-week treatment, the testes of all animals were dissected, frozen immediately in liquid nitrogen, and stored at −80°C for gene sequencing.

To extract total RNA, 200 mg of the testicular sample was processed using TRIzol by following manufacturer (Invitrogen, Carlsbad, CA, USA) protocols and its expression determined using a 2100 Bioanalyzer (Agilent Technologies, Santa Clara, VCA, USA). Only qualified RNAs from testicular samples were used for construction of cDNA libraries. Preparation and sequencing of cDNA libraries were undertaken by the BGI Genomics Co., Ltd. (BGI, Shenzhen, China) using the BGISEQ-500 platform.

To analyze RNA-sequencing data, initially raw reads were excluded if they contained >10% nitrogen, or were adapter or low-quality reads, using SOAPnuke v1.5.2 by BGI. High-quality reads were aligned to the reference genome (mouse) using HISAT v2.0.4 and gene expression was normalized to fragments per kilobase of exon model per million mapped reads (FPKM) using RSEM v1.2.12 by BGI. Normalized FPKM expression was analyzed using Dr Tom v2.0 by BGI to identify differentially expressed genes. The 100 most-regulated genes in the testes of WP-treated OM, and their corresponding gene expression fold-changes in the testes of SB-treated OM, were selected as typical gene signatures to compare the gene profile. The 100 most-regulated TGs consisted of 50 upregulated genes and 50 downregulated TGs. The comparison of gene heatmaps between WP and SB was made by Dr Tom v2.0 by BGI. Besides, Pearson’s correlation analysis was applied to quantitatively analyze the similarity in gene expressions in the testes of OM after oral treatment with SB or WP.

**Spermatogenesis repair by SB**

***Dosing:*** OM were divided into four groups of six and treated with SB (20 mg/kg/d, once daily, i.g.), WP (1.56 g/kg/d, once daily, i.g.), TP (0.2 mg/kg/twice a week, i.p.) or PS (14 mL/kg/d, i.g.), respectively. Normal mice (n = 6) were given PS (1 mL/kg/d, i.g.). All animals were given these agents consecutively for 2 weeks and the observations shown below made.

***Sampling of testicular tissue:*** After 2-week treatment, each mouse was anesthetized with diethyl ether. Tissue from the left testes was harvested, stored in 10% formalin, and paraffin-embedded for staining (hematoxylin and eosin).

***Sperm sampling:*** After 2-week treatment, the limbs of each mouse (under anesthesia) was fixed on a thermostatic hot plate (37°C). The left epididymis was dissected promptly, cleaned with PS (37°C) and transferred immediately to 0.5 g of bovine serum albumin per 100mL of medium 199 (1 mL, 37°C). Tissue was cut into pieces by scissors. Sperm was allowed to flow out of the tissue, and then placed in an incubator in an atmosphere of 5% CO_2_ for 3 min at 37°C. After incubation, the suspension was mixed homogeneously by a pipette, then 10 μL of sperm suspension was placed on a semen-counting slide (Yulu Optics, Nanjing, China). This slide had a depth of 0.01 mm, and enabled unimpeded movement of sperm.

***Microscopic observation of sperm:*** The sperm-counting slide was placed under a phase-contrast microscope (E200; Nikon, Tokyo, Japan). A video was recorded by a semen analysis automatic detection system (Suiplus; Beijing, China). Five visual fields were taken from each counting slide for observation. The movement track, morphology, concentration and number of sperm were observed, and recorded for qualitative evaluations and parameter evaluations.

***Quality parameters of sperm:*** IVOS software (Hamilton Thorne Biosciences, Beverly, MA, USA) in the semen analysis automatic detection system (Suiplus) was used to evaluate the quality parameters of sperm. The parameters were sperm concentration, sperm mobility, progressive mobile sperm, sperm motion velocity (VCL, VSL, VAP), sperm-motion locus (STR, LIN) and dynamic parameters of sperm movement (BCF, ALH).

**Efficacy of SB in enhancing male reproductive ability**

***Dosing:*** OM were divided into four groups of three and treated with SB (20 mg/kg/d, once daily, i.g), WP (1.56 g/kg/d, once daily, i.g.), TP (0.2 mg/kg, twice a week, i.p.) and PS (14 mL/kg/d, once daily, i.g.), respectively. Normal mice (n = 3) were given PS (14 mL/kg/d, i.g.). All animals were given these agent consecutively for 2 weeks.

***Reproductive ability:*** After 2-week treatment, each male mouse was mated with females at a 1:2 ratio. Mating mice were placed in one cage for 10 days (two sex cycles of females). Females were examined for pudendal embolus each morning at 8:30. The plugged female was removed from the cage immediately. If there was no sign of intercourse, the female(s) and male mice were placed in the same cage continuously until the end of the tenth day. After 10 days, female mice were separated from the male mouse, and observed for 40 days. The total number of pups in the first litter for a pregnant female, and the number of non-pregnant females, was recorded. The ANB was calculated using the formula:

ANB = total number of births/number of females who gave birth

**Gene profiling and biologic pathways regulated by SB**

***Dosing and sampling:*** OM were divided into two groups of six and treated once daily with SB (20 mg/kg/d, i.g.) or PS (14 mL/kg/d, i.g.), respectively. After 2-week treatment, each mouse was anesthetized with diethyl ether. Testicular tissue was frozen immediately in liquid nitrogen and stored at −80°C for further analyses. Normal male mice were included as a blank control (n = 6). After experimentation, mice were sacrificed by cervical dislocation.

***Gene profiling and GO analyses:*** Frozen testicular samples (n = 6) from SB-treated or non-SB-treated OM were used for RNA sequencing. Extraction of total RNA and data analyses were done as described above. Furthermore, functional annotation of differentially expressed genes in the GO database was applied using Dr Tom v2.0 by BGI.

**RT-qPCR verification**

Frozen testicular samples (n = 3) from normal mice, OM, or SB-treated OM were used for RT-qPCR. Total RNA was extracted using a TRIzol Plus RNA Purification kit (Invitrogen), and analyzed (excitation wavelength =260 nm, emission wavelength = 280 nm) using a spectrophotometer (Nano300; Allsheng, Hangzhou, China).

cDNA was reverse-transcribed from 1 μg of total RNA using PrimeScript RT reagent (TaKaRa Biotechnology, Shiga, Japan), and 10 ng of cDNA was analyzed using SYBR Premix Ex Taq II (TaKaRa Biotechnology) on a CFX Connect TM Real-Time PCR Detection System (Bio-Rad Laboratories, Hercules, CA, USA). Each sample was tested in triplicate. Glyceraldehyde 3-phosphate dehydrogenase (GAPDH) was used as an internal control. Relative quantification of genes of interest was done using the 2^−ΔΔct^ method. Primer sequences used for RT-qPCR (forward and reverse, respectively) were 5′- TGCTCTTCTGGCGTGCTTCTTG-3′ and 5′- TGTAGTCCTGGTCTTCCTCCTCCT-3′ for the *Fst* primer; 5′- GTCCTCGCTCTCCTTCCACTCAA-3′ and 5′- AGCAGCCACACTCCTCCACAAT-3′ for the *Inhba primer*; 5′- AGAAGGTGGTGAAGCAGGCATCT-3′ and 5′- CGGCATCGAAGGTGGAAGAGTG-3′ for the *GAPDH* primer.

**Pharmacokinetics**

***Working solutions:*** Pure SB was weighed accurately and dissolved in methanol to prepare working standard solutions (0.05–30.0 ng/mL). The IS solution of testosterone (25.0 ng/mL) and arctigenin (25.0 ng/mL) was prepared similarly for SB measurements in plasma and testicular tissue, respectively. All solutions were stored at 4°C before use.

***Sampling of blank plasma and testicular tissue:*** Normal mice (n = 15) were anesthetized with diethyl ether. Aliquots of venous blood (0.75 mL) were sampled, centrifuged at 5000 rpm for 10 min at 4°C to obtain plasma, and stored at −80°C until use. Animals were sacrificed, testicular tissues were collected on an ice plate at the same time of blood sampling, and frozen immediately at −80°C for use.

***Calibration curves and quality control (QC):*** Calibration curves and QC samples for SB in plasma and testicular tissue were prepared in duplicate to evaluate the precision, accuracy, stability and recovery of our analytical method. The handling procedures are described below.

***Plasma handling:*** Plasma was thawed at 4°C for ~30 min and vortex-mixed for 30 s. Plasma (200 μL) was vortex-mixed with 60 μL of a working solution of SB for 30 s, added to 60 μL of IS solution (testosterone) and vortex-mixed for 30 s. Then, 600 μL of acetonitrile was added, followed by vortex-mixing for 120 s, and centrifugation at 13,000 rpm for 10 min at 4°C. The resultant supernatant was injected into the UPLC-MS/MS system. SB concentrations for calibration curves were prepared at 0.05, 0.10, 3.0, 6.0, 12.0 and 25.0 ng/mL in plasma, whereas those for QC analyses were prepared at 1.0, 10.0 and 20.0 ng/mL in plasma. In these samples, the IS concentration was 25.0 ng/mL.

***Handling of testicular tissue:*** Testicular tissue was thawed at 4°C for ~30 min, washed with PS, drained with filter paper and weighed accurately. Then, PS (1:4, *w/v*) was added, and the tissue homogenized. The homogenate (200 μL) was added to 60 μL of SB, and 60 μL of IS (arctigenin) working solution. The mixture was vortex-mixed for 30 s, followed by addition of 400 μL of acetonitrile, vortex-mixing for 120 s, and centrifugation at 13,000 rpm for 15 min at 4°C. The resultant supernatant was injected into the UPLC-MS/MS system. SB concentrations for calibration curves were 0.10, 0.20, 0.50, 1.0, 5.0, 20.0 and 30.0 ng/mL in testicular tissue, whereas those for QC analyses were 1.0, 10.0 and 20.0 ng/mL in testicular tissue. In these samples, the IS concentration was 25.0 ng/mL.

***Analytical conditions:*** After oral administration of SB in male mice, concentrations of SB in plasma and testicular tissues were detected by UPLC-MS/MS. Analyses were undertaken on a UPLC system (Acquity UPLC Ӏ-Class system; Waters) consisting of an auto-sampler, quaternary pump, and a column oven. A C18 reverse-phase column (Acquity UPLC BEH, 100 × 2.1 mm, 1.7 μm, 130 Å) was used to separate samples. The mobile phase comprised 0.1% formic acid in water (A) and acetonitrile (B). Gradient elutions were: 0 min 50% B, 0.5 min 50% B, 1.5 min 80% B, and 6 min 50% B. Samples were kept in the autosampler at 4°C until measurement. The column was maintained at 40°C, the flow rate was 0.3 mL/min, and injection volume was 2 μL. Detection was carried out on a Xevo triple quadrupole mass spectrometer (Waters). High-purity nitrogen served as the nebulizing gas and drying gas. Optimal MS conditions were: positive ion mode, source temperature = 110°C, desolvation-gas temperature = 450°C, cone gas flow = 50 h, desolvation gas flow = 600 L/h, capillary voltage = 3.0 kV, sampling cone voltage = 25 V, and extraction cone voltage = 3.0 V. Multiple-reaction monitoring data were acquired in centroid mode between *m* 50 and *m/z* 1000 using MassLynx v4.1 (Waters), and the scan time and interscan time were set at 0.4 s and 0.1 s, respectively. Leucine-enkephalin (*m/z* 556.2771) was used as the external reference of LockSpray infused at a constant flow of 5 μL/min. The mass spectrometer was calibrated over a range of 50–1000 Da with sodium formate. The following precursors to product ions were monitored: *m/z* 401.2843→300.3354 for SB (collision energy, 24 eV; dwell time, 25 ms); *m/z* 289.4323→253.3991 for testosterone (14 eV; 25 ms); *m/z* 373.3807→355.3415 for arctigenin (48 eV; 25 ms).

***Specificity:*** Blank plasma, blank plasma with addition of working solutions of SB and IS, and plasma samples after oral administration of SB were analyzed by UPLC/MS/MS for exclusion of interference at the peak concentration of SB or IS. Similarly, specificity for measurement of SB in testicular tissue was also validated.

***LLoQ:*** The LLoQ was determined as the lowest concentration that the instrument could quantify accurately (i.e., the lowest concentration point on the standard curve).

***Precision and accuracy:*** The precision and accuracy were validated by measuring QC samples at 1.0, 10.0 and 20.0 ng/mL of SB in plasma (n = 3) or in testicular tissue (n = 3), respectively. During measurements in 3 consecutive days, the intra- and inter-day variations were calculated. Precision was expressed as the relative standard deviation (RSD)% and accuracy was expressed as the relative error (RE)% by comparing the SB concentration measured with the SB concentration added. The criterion for acceptability was: precision, <15%, accuracy, 85%–115%; LLoQ ± 20% accuracy.

***Extraction recovery:*** SB recovery from plasma or testicular tissue was calculated by comparing the SB concentration measured with the SB concentration added.

***Sample stability:*** SB stability was assessed on the QC samples mentioned above at three concentrations after three freeze–thaw cycles (−20°C to 25°C) on 3 consecutive days, storage at 25°C for 24 h, and storage at −80°C for 1 month, respectively. Sample stability was expressed as the RSD for the SB concentration measured.

***Dosing:*** Normal male mice (n = 65) were fasted 12 h but had free access to water. Then, each mouse was administered (p.o.) a single dose of SB (20 mg/kg, i.g.) for subsequent experiments.

***Sampling:*** Blood sampling was done at 0 min (before dosing), 15 min, 30 min, as well as 1, 1.5, 2, 3, 4, 5, 6, 8, 12 and 24 h (five mice at each time point) under anesthesia. Aliquots of venous blood (0.75) were sampled, centrifuged at 5,000 rpm for 10 min at 4°C to obtain plasma, and stored at −80°C until use. After each blood sampling, animals were sacrificed, testicular tissues were collected on an ice plate, and frozen immediately at −80°C for use.

***Plasma handling:*** Plasma was thawed at 4°C for ~30 min and vortex-mixed for 30 s. Plasma (200 μL) was vortex-mixed with 60 μL of IS solution (testosterone) for 30 s, followed by addition of 60 μL of methanol. After vortex-mixing for 30 s, 600 μL of acetonitrile was added, followed by vortex-mixing for 120 s, and centrifugation at 13,000 rpm for 10 min at 4°C. The resultant supernatant was injected into the UPLC-MS/MS system.

***Handling of testicular tissue:*** Testicular tissue was thawed at 4°C for ~30 min, washed with PS, drained with filter paper, and weighed accurately. Then, PS (1:4, *w/v*) was added and the tissue homogenized. The homogenate (200 μL) was added to 60 μL of methanol and 60 μL of IS (arctigenin) working solution. The mixture was vortex-mixed for 30 s, followed by addition of 400 μL of acetonitrile, vortex-mixing for 120 s, and centrifugation at 13,000 rpm for 15 min at 4°C. The resultant supernatant was injected into the UPLC-MS/MS system.

***Pharmacokinetic analyses:*** Pharmacokinetic parameters in plasma and testicular tissue were calculated using a non-compartmental approach employing DAS v3.2 (China State Drug Administration, Shanghai, China).

**Statistical analyses**

Statistical analyses were conducted by Prism v7.0 (GraphPad, La Jolla, CA, USA) and SPSS v20.0 (IBM). No data were excluded from analyses. The Student's *t*-test (two-tailed) or one-way analysis of variance was used for statistical analyses. p < 0.05 was considered significant. Data are the mean ± standard deviation.

**Supplementary information**

All data needed to understand and assess the conclusions of this research are available in the main text and supplementary materials. Raw datasets supporting the findings of this study are available online or from the corresponding author.
