# Supplementary material for: Schisandrin B for the treatment of male infertility
Source: Clin Transl Med. 2021 Feb 23;11(2):e333. doi: 10.1002/ctm2.333 (PMC7901724; doi:10.1002/ctm2.333)
Supplement: Supplementary file 2 — FigureS1–S2 [file CTM2-11-e333-s002.docx]

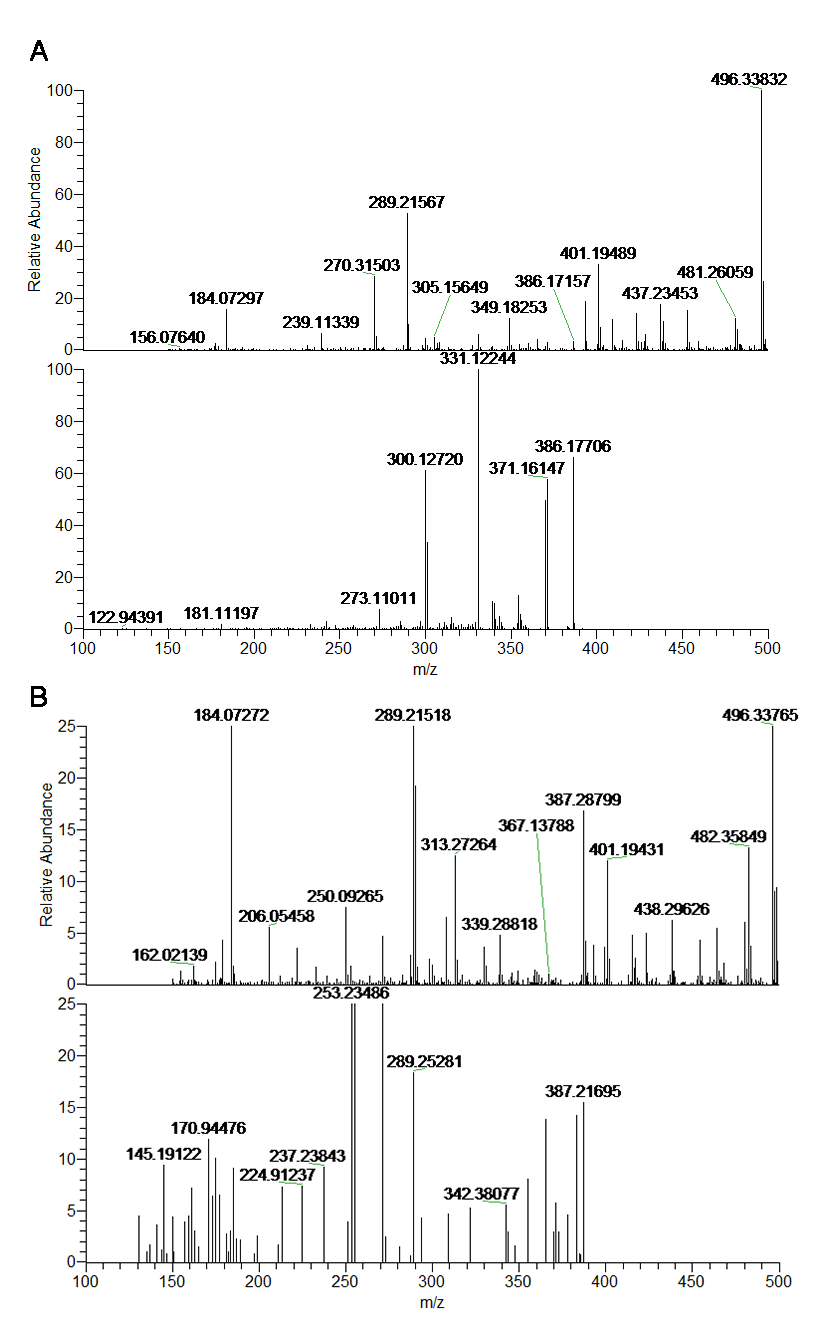


**Fig. S1. Identification of schisandrin B in mouse plasma and testicular tissue**

***Notes:***

**A.** Typical mass spectra of mouse plasma after oral administration of schisandrin B (20mg/kg) at 3 h in Fig.1G, which was used as identifying schisandrin B structure in plasma.

**B.** Typical mass spectra of mouse testicular tissue after oral administration of schisandrin B (20mg/kg) at 3 h in Fig.1I, which was used for identifying schisandrin B structure in testicular tissue.

**
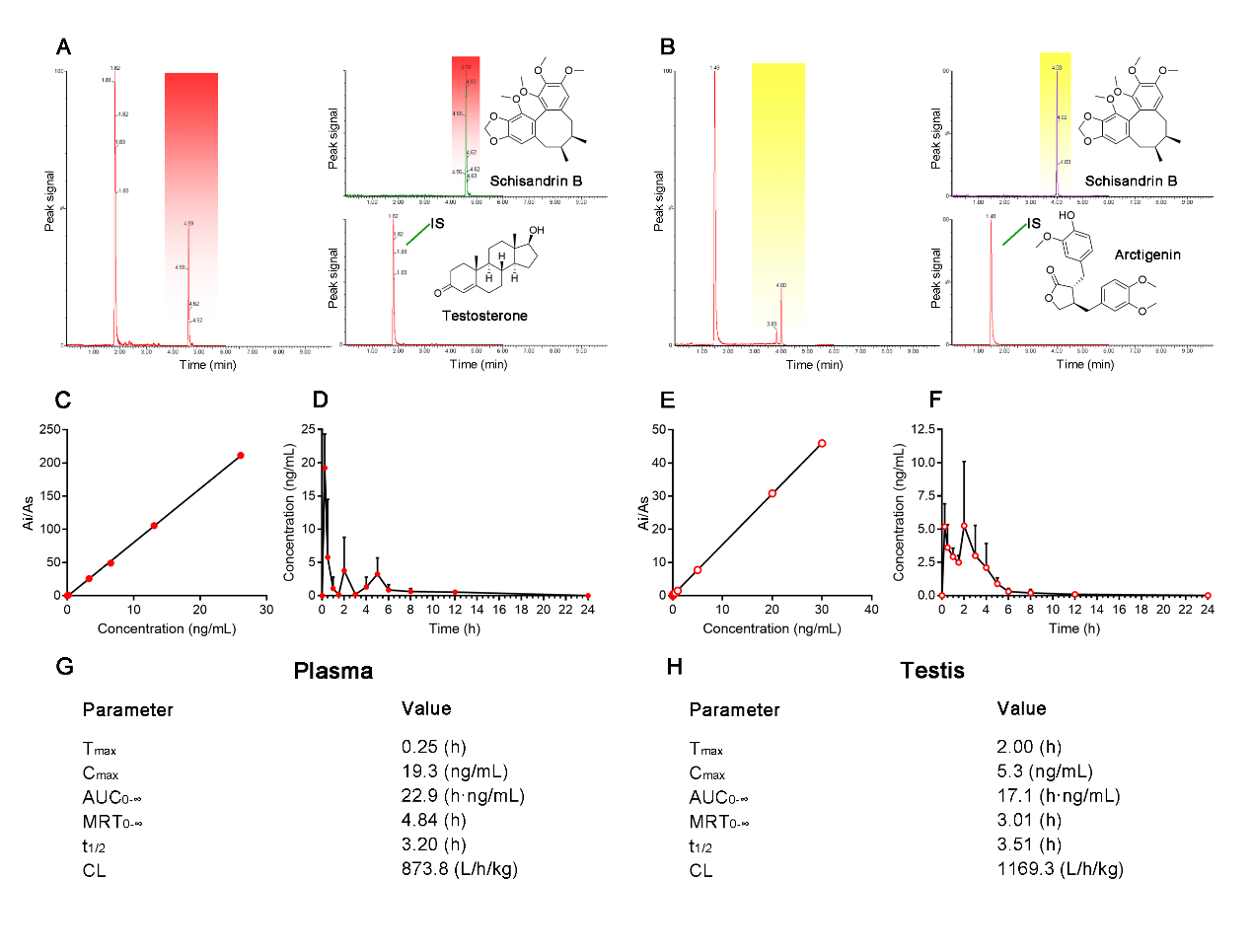
**

**Fig. S2. Pharmacokinetics of schisandrin B in plasma and in testicular tissue in normal mice after oral administration**

***Notes:***

The studies (**A-F**) were performed to establish analysis method for the separation and determination of schisandrin B (SB) in plasma and testicular tissue of normal mice.

**A.** Typical multiple reaction monitoring (MRM) chromatogram of mouse plasma after oral administration of SB (20mg/kg) at 3 h. Internal standard (IS) added in plasma, testosterone. The results display that the chromatogram peaks of SB and IS in plasma appear at 4.59 min and 1.82 min, respectively, and there are no interfering peaks in the chromatogram, demonstrating a good specificity of analysis method for plasma samples.

**B.** Typical MRM chromatogram of mouse testes after oral administration of SB (20mg/kg) at 3 h. IS added in testicular tissue, arctigenin. The results display that the chromatogram peaks of SB and IS in testicular tissue appear at 4.00 min and 1.48 min, and there are no interfering peaks in the chromatogram, demonstrating a good specificity of analysis method for testicular samples.

**C.** Calibration curve of SB in mice plasma. Ai indicates peak area of SB; and As indicates peak area of IS (testosterone) in plasma samples. The results show that the peaks and concentration of SB are linearly correlated in the range of 0.07 to 26.09 ng/mL in plasma.

**D.**  Mean plasma concentration-time curve of SB. The sampling was performed at 0 min, 15 min, 30 min, 1 h, 1.5 h, 2 h, 3 h, 4 h, 5 h, 6 h, 8 h, 12 h and 24h before and oral administration of SB (20mg/kg) (n= 5). The results show that SB can be rapidly absorbed into blood, and has triple absorption peaks, suggesting a hepato-intestinal circulation pathway during absorption and metabolism.

**E.** Calibration curve of SB in testicular tissue. Ai indicates peak area of SB; and As indicates peak area of IS in testicular samples. The results show that the peaks and concentration of SB are linearly correlated in the range of 0.10 to 30.00 ng/mL in testicular tissue.

**F.** Mean testicular concentration-time curve of SB. The sampling was performed at 0 min, 15 min, 30 min, 1 h, 1.5 h, 2 h, 3 h, 4 h, 5 h, 6 h, 8 h, 12 h and 24h before and oral administration of SB (20mg/kg) (n= 5). The results show that SB reaches the testicular tissue rapidly after absorption, demonstrating that SB is able to reach the action site.

The studies (**G-H**) were performed to calculate the pharmacokinetic parameters in mice plasma and in testicular tissues by software of DAS v3.2 (China State Drug Administration, Shanghai, China).

**G.** Major pharmacokinetic parameters in mice plasma after oral administration of SB.

**H.** Major pharmacokinetic parameters in mice testicular tissue after oral administration of SB.
